# A Preliminary Study of Functional Brain Activation among Marijuana Users during Performance of a Virtual Water Maze Task

**DOI:** 10.1155/2013/461029

**Published:** 2012-12-17

**Authors:** Jennifer Tropp Sneider, Staci A. Gruber, Jadwiga Rogowska, Marisa M. Silveri, Deborah A. Yurgelun-Todd

**Affiliations:** ^1^Neurodevelopmental Laboratory on Addictions and Mental Health, McLean Hospital, 115 Mill Street, Mail Stop 204, Belmont, MA 02478-1064, USA; ^2^McLean Imaging Center, McLean Hospital, Boston, MA, USA; ^3^Department of Psychiatry, Harvard Medical School, Belmont, MA, USA; ^4^Cognitive and Clinical Neuroimaging Core, McLean Hospital, Belmont, MA, USA; ^5^The Brain Institute, University of Utah Medical School, Salt Lake City, UT, USA

## Abstract

Numerous studies have reported neurocognitive impairments associated with chronic marijuana use. Given that the hippocampus contains a high density of cannabinoid receptors, hippocampal-mediated cognitive functions, including visuospatial memory, may have increased vulnerability to chronic marijuana use. Thus, the current study examined brain activation during the performance of a virtual analogue of the classic Morris water maze task in 10 chronic marijuana (MJ) users compared to 18 nonusing (NU) comparison subjects. Imaging data were acquired using blood oxygen level-dependent (BOLD) functional MRI at 3.0 Tesla during retrieval (hidden platform) and motor control (visible platform) conditions. While task performance on learning trials was similar between groups, MJ users demonstrated a deficit in memory retrieval. For BOLD fMRI data, NU subjects exhibited greater activation in the right parahippocampal gyrus and cingulate gyrus compared to the MJ group for the Retrieval-Motor Control contrast (NU > MJ). These findings suggest that hypoactivation in MJ users may be due to differences in the efficient utilization of neuronal resources during the retrieval of memory. Given the paucity of data on visuospatial memory function in MJ users, these findings may help elucidate the neurobiological effects of marijuana on brain activation during memory retrieval.

## 1. Introduction

Research on marijuana (MJ) use continues to be a major area of investigation, since MJ remains the most widely used illicit drug in several countries, including the United States [1]. Daily, long-term, and frequent MJ use can have serious adverse effects on mental and physical health and can affect work performance, family, and school functioning [2]. In 2009, epidemiological data (Treatment Episode Data Set) indicated that MJ was associated with 740,800 substance abuse treatment admissions, with daily use being reported in 23% of treatment entries [2]. Nearly half (46.3%) of daily MJ admissions occurred in individuals between ages 26 and 40 years old and 34.2% between ages 18 and 25 years old [2]. In addition, there has been a rise in the prevalence of MJ use among youth, with 36.4% and 22.6% of high school seniors reporting past year and past 30 days use, respectively. Given that rapid brain maturation occurs from adolescence into the early twenties [3, 4], a time when MJ use is often initiated and tends to increase, identifying neurobiological vulnerabilities associated with MJ use is critical.

Short-term effects of MJ have been reported across a number cognitive domains (for review [5, 6]), including deficits in memory [7], attention and mental flexibility [8], response inhibition [9], decision making [10, 11], emotional processing [12], and impulsivity [13]. However, to date there have been limited studies examining the effects of MJ on spatial memory processing. While chronic MJ users have been shown to exhibit deficits on tests of visual recognition, delayed visual recall, and prospective memory [14], a meta-analytic study failed to find substantial long-term neurocognitive deficits, except in the domains of learning and forgetting [15]. To this end, significant structural and functional changes have been reported in young MJ users in brain regions implicated in learning and memory [16–19].

The hippocampus may be notably vulnerable to the effects of MJ, given the high density of cannabinoid receptors in this area [20]. Findings from animal studies have provided evidence supporting cannabinoid-induced impairments on hippocampal-mediated memory tasks [21–27]. For instance, activation of cannaninoid-1 (CB_1_) receptors in mice in the hippocampal region inhibited long-term potentiation (LTP), which is a neurobiological model for learning and memory [28]. Administration of delta-9-tetrahydrocannabinol (Δ^9^-THC) impaired spatial memory in mice tested on the Morris water maze task (WMT), while the CB_1_ receptor antagonist SR141716A reversed the impairment [26]. Similar Morris water maze impairments were reported in mice after injection of Δ^9^-THC or inhalation of marijuana smoke [24]. These findings provide evidence of cannabinoid-induced impairments on hippocampus-dependent spatial learning tasks, likely due to interference in learning acquisition and retrieval processing.

The hippocampus is necessary for processing spatial layout and configural representation of an environment [29–35], as rodents with hippocampal lesions demonstrate spatial memory deficits, evidenced as an inability to find a hidden platform in the Morris WMT [30, 36, 37]. In addition to compelling animal literature indicating that MJ exposure impacts hippocampal function [23, 25, 38], humans with medial temporal lobe damage, including the hippocampus and associated areas, exhibit impaired declarative memory, such that patients have an inability to describe time, place, and meaning of events [39]. Patients with unilateral hippocampal resections demonstrate impaired spatial navigation during performance of a virtual Morris WMT [40]. Studies employing functional magnetic resonance imaging (fMRI) techniques provide evidence for altered brain activation patterns in memory-related processing regions associated with heavy MJ use. Long-term, heavy MJ users exhibit greater widespread blood oxygen level-dependent (BOLD) activation compared to NU during a spatial working memory task after short-term drug withdrawal [41]. In a study using a visuospatial 2-back working memory fMRI task, MJ users and NU exhibited similar task performance; however, MJ users demonstrated greater activation in the inferior and middle frontal gyri, areas associated with visuospatial working memory, and increased activation in the right superior temporal gyrus, an area not typically recruited for visuospatial working memory [42]. The parahippocampal area also plays an important role in spatial memory, namely, allocentric memory processing, especially during viewing of complex scenes with objects and landmarks (e.g., [43–45]). An increased response in the parahippocampal gyrus has been reported for objects at relevant locations (i.e., at decision making points) during an object-location memory task [46], suggesting that neural activation in the parahippocampus is associated with the navigational relevance of an object's location.

The frontal cortex, specifically, the dorsolateral prefrontal cortex, has been implicated in spatial working memory tasks [47, 48]. While MJ effects on spatial working memory have been the subject of several investigations, the objective of the present study was to investigate MJ-related effects on spatial memory (learning and retrieval) to examine differences in neural activation during the performance of a virtual analogue of the Morris water maze task. Based on the work by Jager and colleagues [49], it was hypothesized that MJ users would demonstrate hypoactivity in the hippocampal/parahippocampal region relative to NU participants. Further, exploratory analysis of the cingulate gyrus was performed, given that this region is activated during WMT performance [50], but also frontal-related alterations associated with MJ use have been previously reported [42, 51, 52].

## 2. Methods

### 2.1. Participants

The study sample consisted of ten chronic marijuana (MJ) users (8 males) and eighteen nonusing (NU) comparison subjects (11 males). Participants were recruited through local advertisement and screened by telephone interview to ensure they met criteria for inclusion in the study. All aspects of the clinical research protocol were reviewed and approved by the Institutional Review Board of McLean Hospital (Belmont, MA, USA). After a complete description of the study, participants provided written informed consent. All participants received monetary compensation ($100) for study completion. Participant demographics are presented in Table 1.

To qualify for study entry, MJ smokers had to have smoked MJ a minimum of 2500 times, used MJ at least five of the last seven days prior to the study visit, test positive for urinary cannabinoids, and meet DSM-IV criteria for MJ abuse on the day of scanning. MJ users were asked to refrain from smoking for 12 hours immediately preceding the study visit and were told a urine sample would be collected at the initiation of the study visit, in order to improve compliance. The NU participants reported fewer than 5 lifetime episodes of MJ use and did not use any other illicit substances. Exclusion criteria for all subjects included history of head injury, loss of consciousness, history of organic mental disorder, seizure disorder or central nervous system disease, and contraindications to MR scanning (e.g., pacemaker, aneurysm clips, metallic implants, pregnancy, or claustrophobia). MJ users reported consuming 4.4 ± 4.3 alcoholic beverages per week, while NU participants reported consuming 1.8 ± 2.5 alcoholic beverages per week (*F*(1,26) = 4.2, *P* = .05). Four MJ users reported recent nicotine use (ranging from 1 pack per day; 1 pack every 2 weeks; 1 pack per month; occasional/social use). NU adults did not report any use of nicotine.

The Barratt Simplified Measure of Social Status (BSMSS) was used to measure socioeconomic status (SES) [53]. Clinical interviews were conducted using the Structured Clinical Interview for DSM-IV (SCID; [54]). All participants were free of Axis I diagnosis, except the MJ group, who were required to meet criteria for MJ abuse. Participants completed the Positive and Negative Affect Scale (PANAS; [55]), a 20-item scale measuring positive and negative affects, and the Hamilton Anxiety Scale (HAM-A; [56]), a 14-item scale measuring anxiety level. The Addiction Severity Index (ASI) was used to evaluate substance abuse using a 5-point scale (0 = not at all; 4 = extremely) for questions regarding seven areas in their life that include medical condition, employment, drug use, alcohol use, illegal activity, family/social relations, and psychiatric function [57]. The Marijuana Withdrawal Checklist (MWC), a 12-item scale, was used to assess withdrawal symptom during the early stages of abstinence is a 12-item scale used [58]. Clinical data are presented in Table 1.

All participants provided a urine sample to be tested for amphetamines, barbiturates, benzodiazepines, cocaine, opiates, phencyclidine, and tetrahydrocannabinol (THC) (Triage Drugs of Abuse Panel: Immediate Response Diagnostics, Biosite, San Diego, CA, USA). A positive result for THC confirmed recent MJ use in the MJ group, while a negative result was required for the NU group. Standard laboratory urinalysis assessed an aliquot of the urine sample, which included gas chromatography-mass spectroscopy in order to quantify the level of 11-nor-9-carboxy-delta 9-tetrahydrocannabinol (THC-COOH) and creatinine (Quest Diagnostics, Cambridge, MA, USA). To allow for differences in urinary concentration among the participants, levels of THC-COOH were normalized to urinary creatinine levels.

A measure of general intellectual ability (IQ) was derived using two of the four subtests (vocabulary and matrix reasoning) from the Wechsler Abbreviated Scale of Intelligence (WASI, [59]). Visuospatial ability and spatial perception were assessed using the Mental Rotation Task [60] and the Santa Barbara Sense-of-Direction Scale (SBSOD) [61]. The Mental Rotation Task is a four-minute paper-pencil test in which participants match a target item to two of four rotated versions. One point is given for a correct response, with a maximum score of 24. The SBSOD is a 15-item self-report measure of environmental spatial ability. The questionnaire consists of several statements about spatial and navigational abilities, preferences, and experiences. Subjects circle a number to indicate their level of agreement with each statement using a seven-point scale ranging from “1: strongly agree” to “7: strongly disagree”.

**Figure 1:**
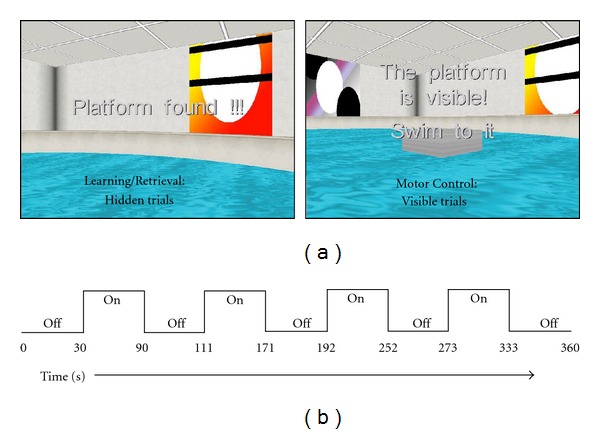
(a) Screen captures of the water maze task during the Learning and Retrieval conditions (hidden trials) (left) and the Motor Control condition (visible trials) (right). (b) BOLD fMRI scanning sequence used during Retrieval and Motor Control conditions.

A PC-compatible laptop was used for testing and operating the virtual water maze program (NeuroInvestigations, Inc., Lethbridge, Canada). The virtual environment was comprised of a circular pool located in the center of a square room, with four large abstract pictures positioned on the walls, which served as landmarks (Figure 1(a)). Subjects viewed the virtual environment from a first-person perspective and navigated through the environment using an MR-compatible joystick that allowed right, left, and forward, but not backward, movements. Participants began each trial facing the wall of the pool, from each of four starting positions: north, south, east, and west. The platform was always located in the northeast (NE) quadrant for all trials for all participants.

Prior to the start of the experiment (nonscanning and scanning conditions), participants completed a training phase outside of the MR suite, which consisted of two trials with the platform visible in the NE quadrant, in order to familiarize them with the task and the use of the joystick. The virtual environment used for training had landmarks that were unique from those in the virtual environment presented during nonscanning and during fMRI. The experimental phase consisted of three conditions: Learning (Hidden Trials—conducted outside the magnet/nonscanning); Retrieval (Hidden Trials—conducted in the magnet); Motor Control (Visible Trials—conducted in the magnet). During the Learning condition, each participant completed 4 blocks of hidden platform trials (4 trials per block, each trial beginning from a different location), in which the platform was hidden under the surface of the water and the participants were instructed to navigate to the platform as quickly as possible. The platform was always located in the same position. Once the participant successfully navigated to the area where the platform was located, a message on the computer displayed “Platform found.” If the platform was not located within 60 sec, the platform became visible and the following message was displayed on the screen: “The platform is visible, swim to it.” The next trial began 1 sec after the previous trial ended (1 sec intertrial interval (ITI)). After completion of the Learning trials, the Probe trial began, in which the platform was removed from the virtual environment unbeknownst to participants. The probe trial ended after participants navigated around the environment for 30 sec.

The Retrieval Condition was similar to the Learning condition and participants were instructed to navigate to the hidden platform as quickly as they could. The platform was always located in the same location as during the learning condition. During the Motor Control condition, each participant completed 2 blocks of visible platform trials (4 trials per block, each trial beginning from a different location). The platform was visibly above the surface of the water and participants were instructed to navigate to the platform as quickly as possible, without paying attention to environmental landmarks. The location of the platform was the same as in the hidden condition, thereby minimizing the potential for encoding novel information during navigation in the environment. The scanning sequence lasted for 360 sec and consisted of alternating “on” (4 active “on” periods) and “off” periods (5 rest “off” periods). During the “on” periods participants navigated through the virtual environment and completed as many trials as possible within each of the four 60 sec “on” periods. Therefore, the number of completed trials varied between participants. During “off” periods, participants viewed a black screen that displayed the message “please wait for instructions” (Figure 1(b)).

Dependent measures for Learning, Retrieval, and Motor Control conditions on the WMT included path length, navigation latency, and first movement latency. Path length (relative to pool diameter) was measured as the distance to reach the platform. Navigation latency was measured as the total time (sec) to complete the task minus the total elapsed time (sec) prior to the first movement. Latency to first movement was measured as the time (sec) before the participant initiated navigation in the pool. Path length (distance to the platform), navigation latency, and latency to first movement measures were averaged across trials per block for the Learning, Retrieval, and Motor Control conditions. For the Probe trial, dependent measures included percent of total distance traversed within the correct platform quadrant (NE), reflecting as an index of spatial learning, and heading error towards the platform, calculated as the angular deviation from a straight path to the center of the platform from the starting position. Heading error was measured at the first occurrence that participant distance was greater than 25% of the pool diameter from the starting position. The number of trials completed during the Retrieval and Motor Control conditions was also recorded.

**Figure 2:**
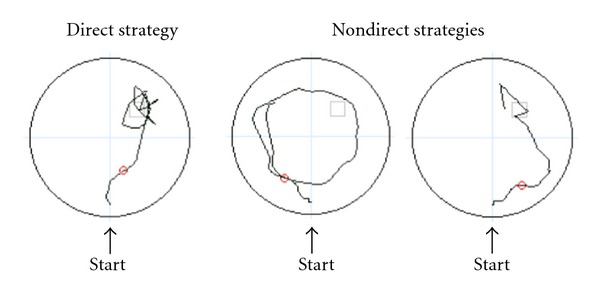
Sample strategies used by participants during the WMT Probe trial. For the direct strategy (left), participants navigated directly to the platform, and for the nondirect strategy (right), participants navigated in a circuitous or random route that was not in the direction of the platform quadrant.

Two independent raters blind to participant diagnosis rated navigation strategies used by participants during the Probe trial. The strategy chosen to solve the water maze could affect behavioral performance [62]. Participant navigation strategies were rated as a direct strategy, where participants navigated directly to the platform location, or a nondirect strategy, where participants navigated in a circuitous or random route that was not in the direction of the platform quadrant (NE) (Figure 2). Interrater reliability for strategy coding was *r* = .53, *P* = .004 (Pearson's *r* correlation coefficient, two-tailed).

### 2.2. Functional MRI Acquisition

Functional MRI scanning was performed on a 3T Siemens Trio whole-body MR scanner (Siemens Healthcare, Erlangen, Germany), using a birdcage quadrature RF head coil for acquisition of echo planar imaging (EPI) blood oxygen level-dependent (BOLD) fMRI. Sagittal scout images were first acquired for alignment and localization using a fast spin echo sequence (FSE) with the following parameters: repetition time (TR) = 3 msec, echo time (TE) = 40 msec, field of view (FOV) = 20 cm, matrix size = 64 × 64, slice thickness = 7 mm (1 mm gap), and flip angle = 90°. Images were acquired from the whole brain using the following parameters: 100 images per slice using a single-shot, gradient pulse-echo sequence, slice thickness = 5 mm, 0 mm skip, flip angle = 90°, TE = 30 msec, and TR = 3000 msec. For each participant, matched T1 and T2 EPI image sets were also obtained: T1-matrix size = 256 × 256, TR = 5760 msec; TE = 80 msec, number of shots = 4, flip = 90°; T2-matrix size = 256 × 256, TR = 6680 msec, TE = 75 msec, number of shots = 4, flip = 90°; 64 × 64 image matrix, 3 mm × 3 mm in plane resolution. In order to maximize the amplitude of the task-induced signal intensity changes, a gradient echo pulse sequence was utilized.The virtual water maze environment was projected via an LCD video projector (Resonance Technology Inc., Northridge, CA, USA) onto a translucent screen located at the rear of the bore, visible to subjects using a mirror mounted on the head coil.

### 2.3. Functional MRI Analyses

SPM5 (Wellcome Department of Imaging Neuroscience, University College, London, UK) was run in Matlab (MathWorks, Natick, MA, USA) for analysis of functional MRI data. To correct for motion in BOLD fMRI data, an intrarun realignment algorithm was utilized, which uses the first image as a reference. An exclusionary criterion of 2 mm of head motion in any direction was used. An EPI template in Montreal Neurological Institute (MNI) stereotactic space was employed to normalize the realigned images, which were resampled into 2 mm cubic voxels. To spatially smooth the normalized images, an isotropic Gaussian filter (full width half maximum [FWHM] = 10 mm) was then applied [63]. In SPM5, high-pass temporal filtering, with a cut-off of 128 sec was applied, and serial autocorrelations were modeled using an AR(1) model. Global scaling was not utilized. Using the framework of the general linear model, statistical analysis for individual subjects was performed [64, 65] using a box-car reference function convolved with the hemodynamic response function.

The motor control condition of the water maze paradigm was used as a control condition since there was no learning or memory component (i.e., subjects simply navigate to the visible platform). Age was entered as a covariate into the analysis. To identify brain areas activated during hidden conditions, predetermined condition effects were calculated at each voxel by the fixed model and a single image of mean activation for Retrieval-Motor Control was created for each subject. The group data were then analyzed using a random-effects model on a second level to account for interindividual variance. Comparisons between groups (NU > MJ; MJ > NU) were performed using a two-sample *t*-test with a priori threshold of *P* < .005, uncorrected, with a minimum extent threshold (*k*) set at 20 contiguous voxels. Anatomic regions (hippocampus, parahippocampal gyrus, and cingulate gyrus) for the region of interest analyses were automatically defined using the Automated Anatomical Labeling atlas [66] in SPM5 using a threshold of *P* < .05 and *k* = 20. Thresholds were based on previously published methods used in BOLD fMRI studies of MJ users [12, 41, 50, 67].

### 2.4. Statistical Analysis

One-way analyses of variance (ANOVAs) were used to compare MJ users and NU on demographic, clinical measures, cognitive measures, and behavioral measures. SPSS 18.0 (SPSS, Chicago, IL) was used for all statistical analyses (*α* = .05). Two-way (Group × Block) repeated measures analyses of variance (ANOVAs) were conducted for path length, navigation latency, first movement latency on WMT Learning trials. One-way ANOVAs were conducted for all other WMT performance measures. Chi-square nonparametric analyses were conducted to compare navigation strategies, that is, direct strategy versus the nondirect strategy, between groups. Significant group differences were observed for age (*F*(1,27) = 5.0, *P* = .03) and education (*F*(1,27) = 5.10, *P* = .03), and, therefore, fMRI analyses included age as a covariate.

## 3. Results

### 3.1. Demographic Variables

As illustrated in Table 1, data for the MJ group confirm near daily MJ use, as indicated by ASI scores of MJ use in the last 30 days, smoking episodes per week, total grams of MJ used per week and average urinary cannabinoid levels (ng/mg). MJ users also reported very low scores (out of a total 36) on the MWC, suggesting no significant withdrawal symptoms were present on study day.

### 3.2. Clinical and Cognitive Measures

No significant differences were observed between the groups for IQ, as measured by WASI, or for mood, as measured by the HAM-A, or on PANAS (positive or negative affect subscales) (Table 2). Further, no significant performance differences were detected between the MJ and NU groups on the Mental Rotation Test Total Score, or on the SBSOD Total Score (Table 2).

### 3.3. Virtual Water Maze Behavioral Performance

#### 3.3.1. Pre-fMRI Hidden Platform Trials: Learning

**Figure 3:**
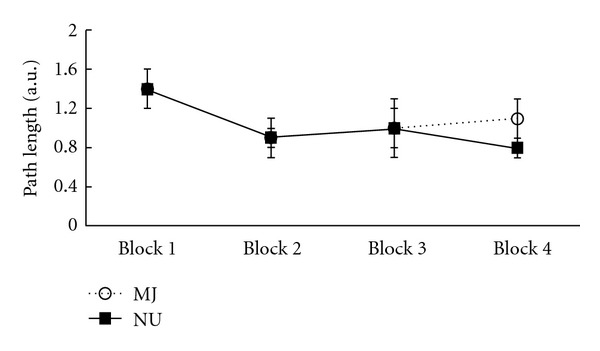
Average path lengths on WMT hidden trials during the Learning condition in MJ (open circles) and NU (closed squares) groups across trial blocks.

There was a significant effect of Block (*F*(3, 78) = 5.1, *P* < .005), with both groups displaying shorter path lengths to reach the hidden platform by the fourth block (Figure 3). For navigation latency, there also was a significant main effect of Block (*F*(3,78) = 3.8, *P* < .05), again with both groups displaying shorter navigation latencies to successfully complete the trial with increasing number of completed trials (Block 1: 16.0 ± 9.2; Block 2: 11.0 ± 6.9; Block 3: 12.9 ± 13.2; Block 4: 9.8 ± 6.4). There was a significant main effect of Block (*F*(3, 78) = 17.1, *P* < .001) for latency to first movement, with both groups displaying shorter latencies to initiate movement by the fourth block (Block 1: 5.6.0 ± 1.8; Block 2: 4.1 ± 1.8; Block 3: 4.1 ± 1.7; Block 4: 3.8 ± 1.9). No interactions Block × Group interactions reached statistical significance for any of these measures.

#### 3.3.2. Pre-fMRI Probe Trial: Retention

The Group effect for Retention trended towards significance, with MJ users displaying a lower percentage of total navigation distance within the NE (correct) platform quadrant on the Probe trial relative to NU (41.6% ± 15.6 versus 50.7% ± 10.6, resp.; include *F* value, *P* = .08). Heading error did not differ significantly between MJ and NU groups (27.9 degrees° ± 28.6 and 20.6° ± 21.5, resp.).

#### 3.3.3. fMRI Hidden Platform Trials: Retrieval

The number of hidden platform trials completed during fMRI did not differ between groups, with MJ users completing 10.3 ± 3.6 and NU completing 11.4 ± 2.7 hidden platform trials during fMRI BOLD acquisition. Despite a lack of difference in the number of trials completed, there was a significant group effect for latency to first movement. MJ users demonstrated shorter latencies to first movement (3.2 sec ± 1.2) relative to NU (4.6 ± 1.6) (*F*(1, 26) = 5.9, *P* < .05) and longer path lengths to reach the hidden platform (MJ: 1.7 ± 1.5 versus NU: 0.8 ± 0.4; *F*(1, 26) = 4.8, *P* < .05). There were no significant group differences for navigation latency (MJ users: 15.1 ± 12.9; NU: 9.9 ± 5.5).

#### 3.3.4. fMRI Visible Platform Trials: Motor Control

The number of visible platform trials completed also did not differ between groups, with MJ users completing 15.7 ± 1.3 and NU completing 15.0 ± 1.4 visible platform trials during fMRI BOLD acquisition. There were no significant group differences observed for path length (MJ users: 0.5 ± 0.1; NU: 0.4 ± 0.01), navigation latency (MJ users: 3.9 ± 0.5; NU: 3.9 ± 0.7), or latency to first movement (MJ users: 3.3 ± 1.4; NU: 4.4 ± 1.7).

#### 3.3.5. fMRI Navigation Strategies

There was a significant preference observed for the use of a direct versus a nondirect strategy during the Probe Trial in the NU group, with 78% (*n* = 14) employing a direct search strategy and 22% (*n* = 4) employing a nondirect strategy (*χ* ^2^(1,18) = 5.6, *P* < .05). A strategy preference was not observed in the MJ group, 50% (*n* = 5) utilized a direct strategy and 50% (*n* = 5) utilized a nondirect strategy.

#### 3.3.6. fMRI BOLD Activation

*Whole-Brain Analysis: Retrieval-Motor Control Condition*. NU, relative to MJ group, demonstrated greater BOLD activation in the bilateral inferior frontal pars triangularis and bilateral inferior frontal pars opercularis, left superior frontal gyrus, left superior frontal pars orbitalis, bilateral middle frontal gyrus, right pallidum, and right putamen for the Retrieval-Motor Control contrast (Table 3). However, the MJ group relative to NU did not show greater BOLD activation in any region (Table 3). 

**Figure 4:**
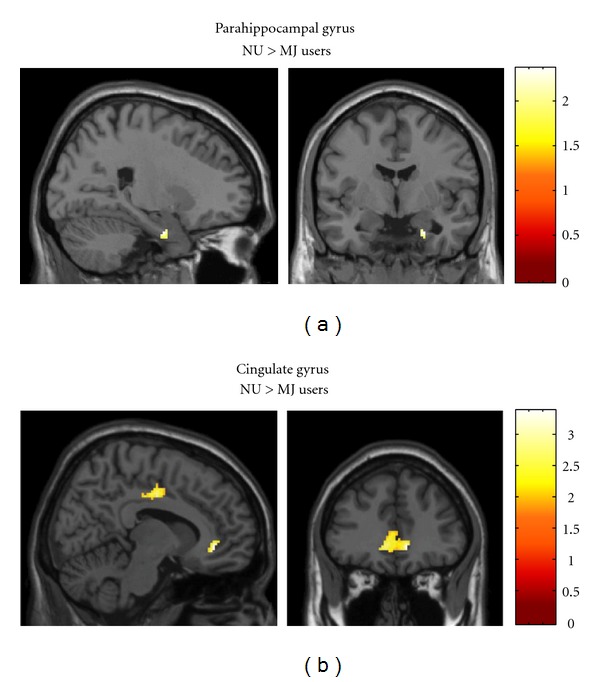
Representative SPM images depicting significant activation for the Retrieval-Motor Control contrast for NU > MJ contrast. (a) NU displayed greater activation in the right parahippocampal gyrus relative to the MJ group. (b) NU displayed greater activation in the bilateral anterior cingulate gyrus and bilateral midcingulate gyrus relative to the MJ group. For the MJ > NU contrast, the MJ group showed no regions of greater BOLD activation relative to NU group. All images are in neurological orientation, that is, left = left and right = right; the color bar at the right reflects the suprathreshold value of the SPM (*t*) statistic for the analysis.

*Region of Interest Analysis: Retrieval-Motor Control Condition*. NU, relative to MJ group, displayed significantly greater BOLD activation in the right parahippocampal gyrus (cluster size (*k*) = 26, *x* = 24, *y* = −4, *z* = −28, *P*  uncorrected = 0.018) for the Retrieval-Motor Control contrast (Figure 4(a)). However, no significant group differences in BOLD activation were detected in the hippocampus. In addition, MJ users demonstrated no regions of greater BOLD activation relative to the NU group in either the parahippocampal gyrus or hippocampus. For the exploratory analysis of the cingulate gyrus, the NU group relative to the MJ group also demonstrated greater bilateral anterior cingulate gyrus and bilateral midcingulate gyrus activation (Figure 4(b)), however, MJ users showed no greater activation in this area relative to the NU group (Table 4).

## 4. Discussion

This pilot study compared current, chronic MJ users and NU during performance of a virtual water maze task of spatial learning and memory. While task performance on learning trials was similar between groups, there was a trend for MJ users to display a lower percentage of total navigation distance within the correct quadrant during the probe trial relative to NU, suggesting a subtle difference in memory retention. During performance on hidden trials during fMRI, although the number of completed trials did not differ between groups, MJ users exhibited significantly longer path lengths and shorter latencies to first movement, which also indicates a deficit in memory retrieval. Performance on visible trials did not differ between groups, however, suggesting that groups had comparable motor abilities. Visuospatial perception (i.e., mental rotation) and environmental spatial ability (i.e., Santa Barbara Sense of Direction Scale) also did not differ between groups, which is consistent with a previous investigation that failed to find differences in orientation skills *in MJ users *[68]. Importantly, this first pilot fMRI investigation of water maze performance in current MJ users revealed that in addition to some behavioral performance differences during memory retrieval, brain activation patterns differed significantly during task performance, with MJ users demonstrating less BOLD activation in the right parahippocampus and the cingulate gyrus relative to NU.

The parahippocampal area is necessary for the processing associations between landmark objects and the environment (landmark-based memory), or contextual memory [69]. In the current study, the NU group demonstrated greater recruitment of the parahippocampus during the retrieval of the hidden platform location, which was located between two relevant landmarks (e.g., abstract paintings) within the environment, which provides consistent support for this brain region being involved in spatial navigation in healthy adults [50]. Although there are no existing data on MJ effects on BOLD activation during a spatial memory task, alterations in hippocampal activation in MJ users relative to nonusers have been reported for nonspatial, associative memory tasks. Jager and colleagues [49] reported that despite normal memory performance, abstinent MJ users displayed hypoactivity in the hippocampal/parahippocampal area compared to NU during the learning phase of a pictorial memory pairing task [49]. In contrast, hyperactivation of the hippocampal/parahippocampal area was reported during the learning phase in a face-name matching task in MJ users compared to nonusers [51]. Similarly, hyperactivation in the left parahippocampal gyrus was observed during the encoding of face-profession pairs in high-frequency versus low-frequency MJ users [70]. Overall, these data suggest evidence for hippocampal/parahippocampal activation differences associated with MJ use during the performance of memory tasks, although memory for objects versus memory for faces may underlie the MJ-related differences in the direction of BOLD activation effects (hypoactivation versus hyperactivation) across these previously published studies.

In the present study, exploratory analysis of the cingulate gyrus revealed greater activation of the anterior cingulate and the midcingulate gyrus during memory retrieval in the NU group compared to the MJ group. The anterior cingulate cortex is an executive region that plays a critical role in modulation of attention, with reciprocal connections with the amygdala, providing support for its role in arousal and motivation [71]. These preliminary data suggest that NU may differentially activate this frontal region to meet the attentional demands posed by this task, which subsequently leads to a greater utilization of attentional resources during memory retrieval as compared to MJ users. These findings are consistent with previous studies, in which frontal hypoactivation was observed in MJ users relative to nonusers [51]. Nestor and colleagues [51] demonstrated hypoactivation in the frontal gyrus in MJ users relative to nonusers during the learning phase in a face-name association task. The majority of remaining available data on brain activation differences associated with MJ use are based on investigations of spatial working memory, which employs a different neural circuitry that is more frontally mediated, compared to more traditional memory tasks that involve the medial temporal lobe. Nonetheless, adolescents with recent MJ use exhibit greater activation of the medial and left superior prefrontal cortex and bilateral anterior insula, despite similar task performance, and as compared to an abstinent group and nonusers during the performance of a 2-back spatial working memory task [52]. In addition, young adult MJ users were reported to demonstrate greater activation in the inferior and middle frontal gyri, as well as the right superior temporal gyrus, an area not typically recruited for visual spatial working memory during the performance of a visuospatial 2-back working memory task [42]. Long-term heavy MJ users also exhibit increased activation in the prefrontal cortex and anterior cingulate, as well as the basal ganglia, compared to non-users, during performance of a spatial working memory task [41]. Taken together, these data suggest that MJ users exhibit altered neural functioning during spatially-related cognitive challenges, with deficits being observed in both frontal and temporal cortices and suggesting evidence for compensatory and adaptive functioning to overcome inefficient activation of the neural network associated.

Brain activation changes associated with MJ use appear to be task specific, with some studies demonstrating hyperactivation, suggestive of increased recruitment, and other studies demonstrating hypoactivation, suggestive of inefficient neural networking [42, 51, 52]. Overall, studies that have reported hyperactivation of brain areas suggest functional compensation and possible neural recruitment of additional brain areas [5]. Alterations in brain activation (e.g., hypoactivation) can also be affected by differences in cerebral blood volume (CBV) and cerebral blood flow (CBF) [72]. Studies using dynamic susceptibility contrast magnetic resonance imaging (DSC MRI) have provided important insight into the effects of marijuana on cerebral hemodynamics, demonstrating that while CBV levels begin to normalize with continued abstinence from marijuana in frontal areas, temporal and cerebellar brain regions show slower CBV decreases [73, 74]. These findings have important implications for understanding the effects of changes in the microvasculature blood volume and/or blood flow that can affect fMRI BOLD signal in chronic marijuana users and nonusers [73, 74].

At least in the current study, the strategy chosen to solve the water maze could have affected behavioral performance [62]. A significantly greater percentage of NU than MJ users employed a direct navigation strategy, which relies on spatial cues and is the most efficient means to reach the platform quadrant. Indeed, only half of the MJ users used a direct approach to complete the task, which may have contributed to the trend for worse performance on the Probe trial and greater path lengths during retrieval. It is plausible that choosing a less efficient strategy to solve the water maze could have likewise contributed to differences in BOLD activation during spatial navigation. These preliminary findings should therefore be replicated in a larger sample of MJ and NU subjects, which would permit the ability to examine strategy choice in relationship to BOLD activation during spatial memory task performance.

There are a number of strengths and weakness associated with this pilot study. A strength of this study is that it is the first investigation of hippocampal brain activation during the performance of a virtual analogue of the well-established Morris water maze task in MJ users, who were well characterized, clinically diagnosed with marijuana abuse, and who did not meet criteria for any other substance abuse disorders. Furthermore, self-report of marijuana use was confirmed by urine drug toxicology screen and results from the SCID and the MWC suggest that in this sample of chronic, heavy MJ smokers, they were not experiencing significant withdrawal symptoms at the time of assessment. In terms of limitations, only a modest number of subjects were examined in this pilot study, which limits generalizability and precludes the ability to examine sex differences within the groups. Performance measures of spatial ability typically have large variability and, therefore, the current investigation should be replicated using a larger sample of subjects. However, despite the modest sample size, significant BOLD activation differences in the parahippocampal and cingulate region were detected. Although differences in age may have been a potential confound, fMRI analyses were corrected for age and differences remained significant between groups. The MJ group also endorsed more alcohol and tobacco use than the NU comparison group, albeit at modest, nonclinical levels. Nonetheless, effects on BOLD activation cannot be ruled out, particularly since nicotinic alpha-7 acetylcholine receptors are highly distributed in the hippocampus [75] and alcohol use has been reported to block the induction of long-term potentiation in hippocampal rat slices [76]. Although subjects from the current study were well characterized with regard to their clinical, demographic and MJ-use status, formal personality testing was not completed with instruments designed to specifically assess Axis II pathology, which may impact neurocognitive performance. It is of note, however, that the majority of these subjects (80%) were enrolled in a separate, multiweek study, which repeatedly assessed clinical state through clinical scales and interviews, and none appeared to meet criteria or demonstrate any symptoms of a personality disorder [67, 77]. In terms of fMRI data processing, the Automated Anatomical Labeling (AAL) atlas was used to define region of interest (ROIs). It is difficult to localize activation in small areas such as the hippocampus, since areas such as the parahippocampal cortex, fusiform gyrus, and lingual gyrus surround this area. Thus, the atlas map chosen for analysis could have contributed to varied results across studies.

The length of the period of abstinence from marijuana use has been shown to impact performance on memory tasks and brain function [78]. Even though, the MWC suggests that the MJ users are not experiencing significant withdrawal symptoms, it cannot be discounted that the observed group differences could still reflect residual effects of MJ use and may account for the disparate findings amongst studies. While the MJ users in the Jager et al. study [49] were abstinent for at least 7 days prior to testing, participants in the current pilot study abstained for only a minimum of 12 hours. Regardless, both studies demonstrated hypoactivation of the hippocampus and parahippocampal gyrus in MJ users relative to nonusers during a learning and memory task. In order to examine the potential adverse effects of MJ use on neural activity beyond a week of abstinence, a logical next step to this work would be to examine subjects who have undergone a longer period of abstinence to explore whether functional changes in BOLD signal between MJ users and NU result from potential washout effects associated with drug abstinence.

In summary, data from this pilot study demonstrate significant differences in BOLD activation in MJ users compared to a NU comparison group during memory retrieval on a spatial navigation task. These data suggest that MJ users utilize neuronal resources in a manner that differs from NU, as suggested by the observed hypoactivation of the/parahippocampal area during navigation, but perhaps also from frontal hypoactivation due to the attentional demands of the task. Further research is warranted to determine the potential mechanism of action by which MJ use may affect brain activation during memory retrieval. Nevertheless, the current findings demonstrate that MJ use exerts a significant effect on neural activity, which is relevant to public health concerns associated with understanding the long-term consequences of chronic marijuana use on brain function in young adults. Indeed, altered brain function in the absence of gross behavioral performance differences may be an early indicator of future long-term consequences associated with continued use, particularly given that relatively short history of MJ use in the current study sample. Early alterations in neuronal function may potentially be related to the later manifestation of MJ-related cognitive impairments, as well as an increased risk for psychiatric conditions [79], which underscores the need for additional investigations focusing on the neurobiological consequences of MJ use.

## Figures and Tables

**Table 1:** Demographic and marijuana use data.

	MJ (*n* = 10)	NU (*n* = 18)
Age (years)	20.3 ± 3.6	22.8 ± 5.0
	(Age range: 18–30)	(Age range: 18–33)
Education (years)	13.4 ± 1.5	15.5 ± 2.4
Ethnicity (Caucasian/non-Caucasian)^a^	9/0	11/7
SES^b^	43.4 ± 10.6	49.1 ± 12.2
Age of MJ onset	15.6 ± 1.2	—
Smokes per week	10.7 ± 5.5	—
Grams per week^c^	4.8 ± 4.9	—
Duration of use (yrs)	4.0 ± 2.4	—
ASI (MJ use out of 30 days)	25.5 ± 4.0	—
MWC	2.0 ± 2.1	—
THC (ng/mL)	193.5 ± 219.2	—

Data represent mean ± standard deviation. MJ: marijuana; NU: nonusers; SES: Socioeconomic status; ASI: Addiction Severity Index; MWC: Marijuana

Withdrawal Check List. ^a^One missing data point for MJ user. Non-Caucasian

classification consisted of Asian, African American, and others.

^b^One missing data point for NU. ^c^Two missing data points for grams per week.

**Table 2:** Clinical and cognitive measures.

	MJ (*n* = 10)	NU (*n* = 18)	*F *	*P *
PANAS				
Positive affect	30.9 ± 6.6	33.9 ± 7.5	1.1	0.29
Negative affect	12.2 ± 3.2	11.7 ± 2.4	0.2	0.66
HAM-A	2.7 ± 2.3	1.5 ± 1.7	2.5	0.12
Mental rotation total score	16.6 ± 4.6	14.7 ± 4.9	1.0	0.33
SBSOD total score^a^	4.8 ± 0.7	4.6 ± 1.0	0.2	0.68
WASI IQ	121.3 ± 8.3	120.2 ± 8.9	0.1	0.74

Data represent mean ± standard deviation. MJ: marijuana; NU: nonusers.

PANAS: positive and negative affect scale; HAM-A: Hamilton Anxiety

Scale; SBSOD: Santa Barbara Sense-of-Direction Scale; WASI: Wechsler

Abbreviated Scale of Intelligence. ^a^MJ = 8; NU = 14.

**Table 3:** Foci of maximally activated brain regions—Retrieval-Motor Control.

Whole brain	MNI coordinates				Cluster size (*k*)	*T*-max	Voxel *P* uncorrected	Cohen's *d *
Region	BA	*x *	*y *	*z *				
NU > MJ								
L inferior frontal pars triangularis	45	−46	20	12	44	4.46	<.001	1.78
L inferior frontal pars opercularis								
L superior frontal pars orbitalis	47	−26	46	−2	36	3.78	<.001	1.51
L superior frontal gyrus								
L middle frontal gyrus	46	−42	40	28	201	3.67	.001	1.47
L inferior frontal pars triangularis								
R pallidum	48	18	6	−4	25	3.42	.001	1.37
R putamen								
R inferior frontal pars triangularis	47	40	42	0	25	3.24	.002	1.30
R inferior frontal pars orbitalis								
R middle frontal gyrus								
MJ > NU		—	—	—		—	n.s.	

L: left hemisphere; R: right hemisphere. BA: Brodmann area. *P* < .005 (uncorrected).

**Table 4:** Region of interest analysis of bold activation—Retrieval-Motor Control.

Region	MNI coordinates			Cluster size (*k*)	*T*-max	Voxel *P* uncorrected	Cohen's *d *
	*x *	*y *	*z *				
NU > MJ							
Hippocampus	—	—	—		—	n.s.	
R. parahippocampal gyrus	24	−4	−28	26	2.20	.018	0.88
Anterior cingulate gyrus	10	38	−4	316	3.11	.002	1.24
Midcingulate gyrus	12	−12	40	563	2.97	.003	1.19
MJ > NU							
Hippocampus	—	—	—		—	n.s.	
Parahippocampal gyrus	—	—	—		—	n.s.	
Cingulate gyrus	—	—	—		—	n.s.	

L: left hemisphere; R: right hemisphere. *P* < .05 (uncorrected).
